# The critic’s voice: On the role and function of criticism of classical music recordings

**DOI:** 10.3389/fpsyg.2022.925394

**Published:** 2022-09-29

**Authors:** Elena Alessandri, Antonio Baldassarre, Victoria Jane Williamson

**Affiliations:** School of Music, Lucerne University of Applied Sciences and Arts, Lucerne, Switzerland

**Keywords:** music review, music recording, classical music, expert judgment, performance value

## Abstract

In the Western classical tradition music criticism represents one of the most complex and influential forms of performance assessment and evaluation. However, in the age of peer opinion sharing and quick communication channels it is not clear what place music critics’ judgments still hold in the classical music market. This article presents expert music critics’ view on their role, function, and influence. It is based on semi-structured interviews with 14 native English- and German-speaking critics who had an average of 32 years professional activity in classical music review. We present the first visual model to summarize music critics’ descriptions of their role and responsibilities, writing processes, and their influences (on the market and on artists). The model distinguishes six roles (*hats*): *consumer adviser*, *teacher*, *judge*, *writer*, *stakeholder*, and artist *advocate*. It identifies core *principles* governing critical writing for music as well as *challenges* that arise from balancing the above six responsibilities whilst remaining true to an implicit code of conduct. Finally, it highlights the factors that inform critics’ writing in terms of the *topics* they discuss and the discursive *tools* they employ. We show that music critics self-identify as highly skilled mediators between artists, producers and consumers, and justify their roles as judge and teacher based on a wealth of experience as against the influx of pervasive amateur reviews. Our research approach also offers occupation-based insights into professional music review standards, including the challenges of maintaining objectivity and resisting commercial pressures. This article offers a new viewpoint on music critics’ judgments and recommendations that helps to explain their expectations and reflections.

## Introduction

This article explores the performance evaluation discourse and its context through the examination of the nature and role of one of the most complex and historically relevant authorities in this domain: professional music criticism. The landscape of critical discourse on art criticism – and music criticism within it – dates back to the 19th century, with seminal works by, e.g., Brendel (1855, pp. 231–240), Buck (1905), Hellouin (1906), Calvocoressi (1923), Newman (1925), Fox Strangways (1938/1939), French (1948), Becker (1965), Aschenbrenner (1981), Cone (1981) or Ellis (1995). In the past few decades, this theoretical reflection has been expanded through systematic examinations of specific features and conditions of criticism that cover culture and the arts, including surveys on the status, role and function of classical music critics (e.g., Eatock, 2004; McGill et al., 2005; Kristensen and From, 2015a; Verboord and Janssens, 2015). Within this research focus, art critics have been described as “journalists with a difference” (Forde, 2003, p. 113) and as “journalist with that little something extra,” (Harries and Wahl-Jorgensen, 2007, p. 623). They deal with “culture” as encapsulated in and expressed through “works and practices of intellectual and especially artistic activity” (Williams, 1985, p. 90) Therefore, art, and specifically, music criticism is broadly held to be a “cultural” and not a “literary” practice in the emphatic sense of the concept (Eagleton, 1984, p. 18) – actually an overly intimate relationship with literature as was broadly practiced in the nineteenth century (e.g., Plantinga, 1967, pp. 59–78; Dahlhaus, 1971, p. 12; Dahlhaus, 1981; Schmitz-Emans, 2015) is considered a dangerous liaison (Kramer, 1989), even against the current popular opinion that “[music] criticism is supposed to be the effort of literary, entertaining, and provocative craftsmanship” (Frederik Hanssen in Diederichs-Lafite, 1996, p. 505). Consequently, critics are regarded as “cultural mediators and gatekeepers” (Janssen and Verboord, 2015) or as “cultural intermediary,” to apply a concept coined by Bourdieu (1984), p. 325), defined as, among others, “critics of ‘quality’ newspapers and magazines and all the writer-journalists and journalist-writers’, who have assigned themselves the role of divulging ‘legitimate culture’ ” (Bourdieu, 1984, p. 326). In the wake of Bourdieu’s notion, scholars have analyzed criticism to study the constructs through which music is made meaningful by the “quality press” (Shrum, 1991; Cheyne and Binder, 2010).

In music criticism the aforementioned “little something extra” concerns “the intellectual activity of formulating judgments on the value and degree of excellence of individual works of music, or whole groups or genres” (Bujic, 2011). The basis of such activity is “aesthetic appreciation,” however music criticism encompasses much “more than spontaneous liking”; it assumes the ability “to judge and to talk about style, technique, originality” thus identifying music critics as “experts” in the state of the art (Barzun, 2001, pp. 71–72). In addition, since the early days of music criticism, critics significantly contributed to a collective knowledge (Becker, 1982) that built the parameters upon which current music reviewers seek to analyze the quality and value of a classical music recording. The practice of talking, evaluating, and judging cultural objects as music is, however, culturally determined, i.e., the institutional embeddedness of music criticism is not a minor or marginal issue but rather a central analytical dimension worthy of examination (Blank, 2007).

The music critic’s product, i.e., music criticism or music reviews, is a well-established practice in the history of Western classical music (Schenk-Güllich, 1972; Kirchmeyer, 2017; Dingle, 2019a). In the 18th century music criticism developed into a professional, and, from the 19th century onward, an influential intellectual practice within the European musical discourse (Stuckenschmidt, 1965; Baldassarre et al., 2022). It is important to point out that – starting from early approaches (Mattheson, 1722–1725; Scheibe, 1737/1740) – music criticism was first and foremost developed into a critique of works and compositions (Dahlhaus, 1971; Monelle, 2002), for which Schumann (1854/1985); see also Plantinga (1967) and Hanslick (1870) provide prime examples, rather than an explicit critique of musical performance (Ertelt and von Loesch, 2021). Only “opera criticism offers a striking exception” in this context given its predominant focus on the quality of “opera singers’ voices” (Abbate, 2004, p. 508; see also Fenner, 1994; Baldassarre, 2009; Ellis, 2012).

Genuine performance criticism did not emerge until mid/late-nineteenth century, influenced by a modified understanding of the musical artist’s persona as shaped by the nineteenth-century concept of and discourse on musical virtuosity (Samson, 2003; Gooley, 2009; Ruprecht, 2013; Strandberg, 2014; Stefaniak, 2016; Doran, 2020). The belated recognition of the music performer’s accomplishments is hardly surprising in view of the generally wide-spread dismissive and neglecting stance of music critics toward the role and function of the musical performer that persisted till the beginning of the 20th century. For instance, the famous music critic William James Henderson stated that “the consideration of the performer is the last important office of real criticism; but unfortunately, it is the one on which the public lays the largest attention” (Henderson, 1915, p. 75). During the first half of the 20th century, driven by the innovation in the recording technology (Benjamin, 1980; Siefert, 1984, pp. 114–115; Katz, 2004; Burgess, 2014) and the strengthening of a canon of both the classical music repertoire (Hamer, 2019) and of its auditory appropriation (Nikolsky, 2012; Thorau and Ziemer, 2019), not only was the performer’s reputation as an essential agent significantly enhanced, but also a new form of music criticism developed, focussed on recorded music as the result of the interpreter’s performative choices (Dingle, 2019b): professional reviews of classical music recordings.

Recording criticism is a complex form of reasoned evaluation that is very different from live performance criticism in terms of its text content, process, and purpose (Schick, 1996, pp. 153–165). During the course of the century, recording reviews started to appear regularly in specialized magazines such as *The Gramophone* in the United Kingdom (from 1923 to present), the US-based *American Record Guide* (founded in 1935) and *Fono Forum* (from 1957 onward) in Germany, and soon, from the 1920s, music recording criticism “became commonplace” (Dingle, 2019b, p. 253), i.e., a familiar form of written response to music, which entails the description, analysis, categorization and evaluation of music with a focus on topics linked to music performance (Carroll, 2009; Alessandri et al., 2016a). These critical writings have potential purpose and impact beyond historical record and reader information; they are supposed to influence consumer choices and affect musicians’ careers and the standing of recording labels (Pollard 1998; Alessandri et al., 2014). The significance of music performance criticism can hardly be overestimated given the fact that most of the music people listen to is first and foremost in a recorded format (Elste, 2009).

Previous work by the authors (Alessandri et al., 2015, 2016a, 2016b), in which hundreds of published recording reviews were text-analyzed, offered a first structured model of the content of reviews of classical music recordings. It showed how the evaluation of music performance lies at the core of critics’ writings: the nuanced variety of metaphorical and technical descriptors of the performed sound covers on average over half of the review text and is used by critics to ground and support their judgments of value. Those judgments assess the aesthetic qualities of the performance, but also go beyond that to evaluate the musical output as the result of the artist’s achievement and its importance in the wider music market. This work offered us a solid understanding of the topics discussed in published reviews, but not into the critics’ intentions and motivations in writing.

Building on this analysis of published review content, in the present study we thus expanded this modelling approach from the written word to the spoken dialogue. Through a series of purpose built semi-structured interviews, we sought classical music critics’ opinions in order to understand the motivations and perceived roles behind their self- and situational-descriptions, as well as the narratives they use to justify their methods and compartmentalize their professional identities. This approach allowed us for the first time to move beyond the published critique and contrast critics’ intentions about critique with their actual written outputs.

Research in this area is timely given the, for decades now, repeatedly cited ‘crisis’ regarding a sharp decline not only in music criticism but in all form of art journalism (Boenisch, 2008; Kristensen, 2010; Caduff, 2014; Jaakkola, 2015; Heikkilö et al., 2017; Melnyk, 2019; Widholm et al., 2021) and, not least, also due to the new music consumption behaviors and peer-communication channels in the digital age (Varriale, 2012; Hracs et al. 2016; Baldassarre and Alessandri, 2022). Digital technologies have revolutionized the way we listen to and discuss music, giving artists more direct access to their audiences, creating platforms for peer-opinion, and empowering listeners with new means and resources to facilitate decision making with regards to purchasing and listening (Carboni, 2012; Datta et al., 2017). In a world of peer-opinion, it is reasonable to question the role of professional critics. And yet for the listener, the ease of access to digitalized music, combined with its dematerialization and the displacement of product-ownership (due to streaming services) have combined to create a sense of disconnection to artists and a renewed interest in gathering knowledge about the music and the musicians behind it (Crossley and Bottero, 2015; Arditi, 2018; Hesmondhalgh and Meier, 2018).

To understand the critics’ rapidly changing role in the news pantheon – with regard to which Caduff (2014) wonders whether these changes could really be taken as symptoms of decline or whether they are more likely signs of a re-formation of music criticism – we must scrutinize their place in the classical music market as agents in the cultural industries (Debenedetti, 2006), where they seem to face increasing marginalization from alternative reviews and commercial pressures such as online rating systems, PR stunts, and the influences of ‘celebrity’ classical music artists and fan culture. In the face of this shift, this article focuses on how music critics themselves view their role in today’s classical music market, how they value their professional standards, and how they experience and assess the relationships with artists, music producers and the readers of music critique.

## Materials and Methods

### Participants

We interviewed eight English- and six German-speaking music critics based in UK, Germany, and Switzerland at the time of the study. The critics were recruited *via* social media, radio stations, and specialized communities and all of them had at least five-year experience in reviewing recorded classical music. Besides their extensive practice with record critique, we set no further criteria in terms of quality of their experience, preferring instead to take a wide sample of music critics from print and broadcast media and, for the first time to our knowledge, from different countries (UK, Germany and Switzerland). The fourteen critics (2 women, 12 men; age average 59.14, range 32–76) had an average of 31.71 years activity in major classical music review outlets (range 5–50 yrs) including BBC Music, Gramophone, FonoForum, and Rondo (see Supplementary material 1 for details on the experience of each critic). The gender distribution within the sample reflects the current market, with a clear predominance of male critics (McGill et al., 2005; Reus and Naab, 2014; Reus and Müller, 2017). All had a graduate or postgraduate degree in an art or language related field (7 musicology, 2 German language, 2 music, 1 drama/theatre, 1 English literature, 1 French/German literature). By the time of the interview, our critics had published an average of 40 classical music-recording reviews in the past 12 months. They also had extensive experience as performers, editors, and/or record producers.

The critics completed an online survey prior to their interview in order to collect demographic information and their Goldsmith Musical Sophistication index score (GoldMSI). The GoldMSI is a standardized self-report inventory that measures ability to engage with music in a nuanced, flexible, and effective way (Müllensiefen et al., 2014). As expected, all critics scored far above the population average on this scale (population average: 81.58; music critics: 102.79; range 90–120).

### Interview

In-depth, semi-structured interviews were conducted in United Kingdom, Germany, and Switzerland. This form of interview has been described as “conversations with a purpose” (Legard et al., 2003, p. 138) that explores a person’s opinions, feelings and beliefs. The interviews are structured around a leading thread of discourse based on the main themes of enquiry (in this case the nature, role, and influence of music criticism) while allowing conversation to remain flexible, in terms of topic order and new, unexpected topics raised by the interviewee. This method is an ideal way to collect rich data from a small pool of experts (Harries and Wahl-Jorgensen, 2007).

Interviews focused on: (i) the aspects of a recording that were typically reviewed; (ii) the way these aspects are discussed, in terms of language and rhetorical devices and; (iii) the role of professional music criticism in the classical recording market and its influence on key stakeholders such as artists, music producers and the reading public. The development of the interview schedule followed the results of previous work on published music reviews (Alessandri et al., 2014, 2015, 2016a,b). Themes and hypotheses that emerged from these analyses were used to develop questions and prompts. For example, the extended used of comparative judgments evidenced in the analysis of Gramophone reviews (Alessandri et al., 2014, 2015) gave rise to the prompt “*How important is it to compare the recording reviewed with other recordings?*” (for the full interview schedule see Supplementary material 2). Interviews lasted on average 1 h 42′ (range 1 h 12′ – 2 h 57′). The conversations were audio recorded and transcribed verbatim. The interview protocol was approved by the authors’ university ethical review board. All critics gave written informed consent in accordance with the Declaration of Helsinki.

### Analysis

We used a double-coder inductive thematic analysis, in line with general thematic applied analysis methods (e.g., Guest et al., 2012), to produce a visual map of the topics discussed. The protocol followed Williamson et al. (2012), Williamson and Jilka (2013), and Alessandri et al. (2015, 2016a), with the addition of a third coder and a two-stage procedure to account for the bilingual data (English, German). Three researchers (the authors) were involved; each interview was analyzed by one native speaker (third and second authors) plus one researcher (first author) fluent in both languages, thereby assuring methodological continuity and coherence.

The eight English interviews were analyzed first. The first and third authors examined the transcripts independently using line-by-line open coding, comparing and contrasting quotes and organizing codes to develop a map of emergent themes. These themes were then compared between the researchers. To minimize subjectivity, each researcher in turn explained a theme, justifying it by means of quotes and proposing a definition. Based on the newly developed codebook, all data were re-coded by the two researchers independently. Text parsing in the second stage of coding was performed at minimum close level and multi-layered coding was avoided. If a text fragment encompassed more than one theme, then (i) new ideas were prioritized and (ii) the text was coded for the most salient idea. Avoiding multi-layered coding meant that we could not account for the intricacy of language (e.g., the distance between themes as a proxy to links between concepts). However, this approach allowed us to extract the thematic content in its purest form without being burdened by the nature of language construction and thereby to develop a general model from both an English and a German sample.

The model was then applied to the six German interviews. At this stage the second author, a native German speaker, joined the analysis. Again, all interviews were analyzed independently using the developed theme codes, revising and clarifying definitions where needed. NVivo version 11 was used for the application of codes and for computing agreement level in both stages. Reliability in the application of codes between researchers was high for both English (ƙ = 0.976) and German (ƙ = 0.959) interviews. This protocol permitted a structured development of the final visual model. It also allowed us to test the applicability of the model to a different critique sample, in a different language, with a different musical tradition, and with a new coder.

## Findings

**Figure 1:**
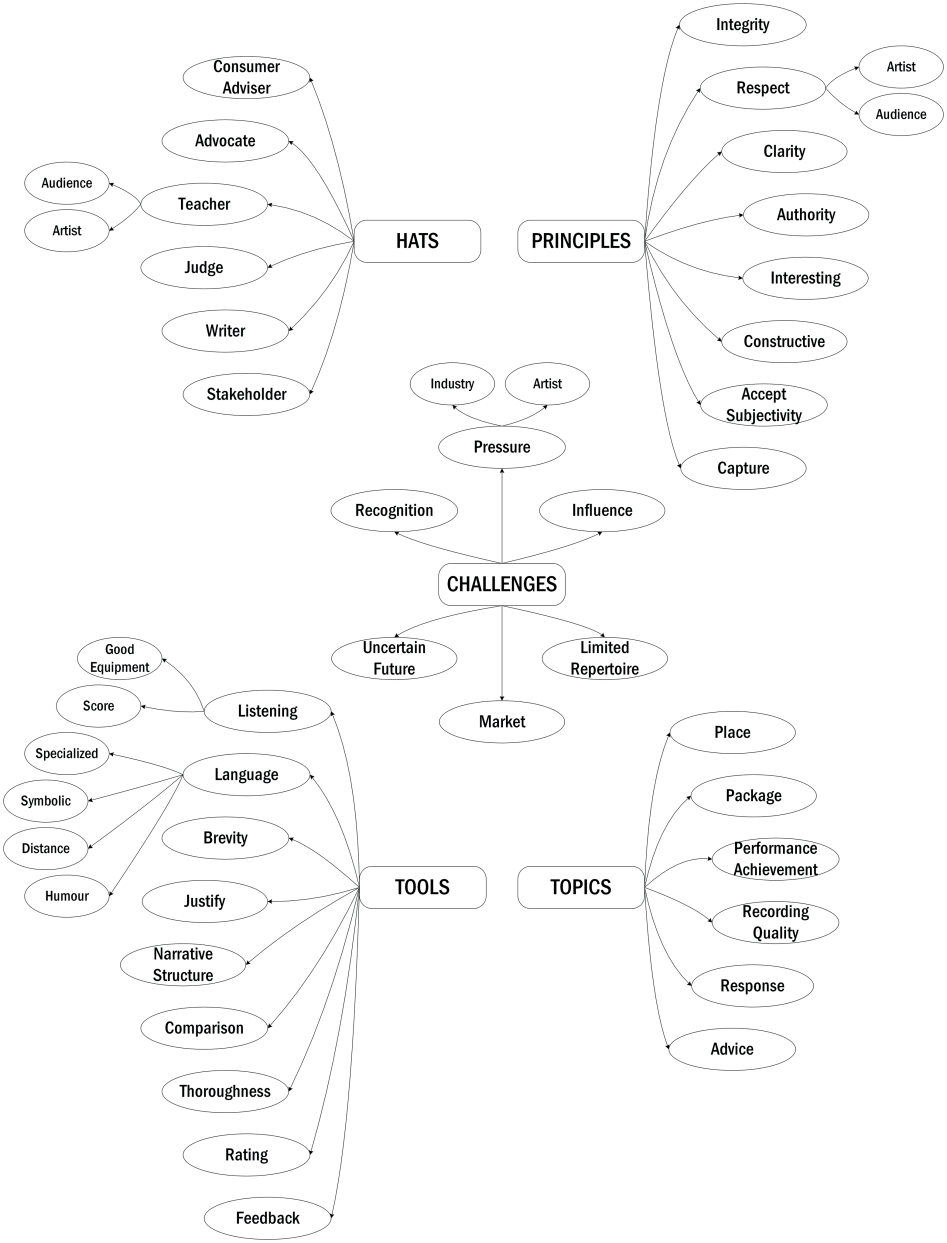
Visual model of the themes discussed by critics. Themes are organized hierarchically from rounded rectangles to ovals; arrows reinforce the visualization of this hierarchical structure.

Five main theme categories emerged from the interviews, which contained a total of 47 themes and sub-themes (Figure 1). Together they described the nature of music criticism through the eyes of critics in terms of their role (**Hats**, **Principles**, and **Challenges**) and strategies (**Topics** and **Tools**). Supplementary material 3 shows all themes with their definitions and example quotes from the interviews. For German quotes, English translations are provided: original German quotes are reported in Supplementary material 4.

### Hats – Things I am

In this first theme category, critics described how they see their role in the classical market. The theme family is called ‘Hats’ to emphasize that critics move between different functions and responsibilities. In interview, they distinguished between six different roles.

Three roles reflect functions usually attributed to cultural intermediaries, mediators or gatekeepers (Bourdieu, 1984; Cheyne and Binder, 2010; Smith Maguire and Matthews, 2012; Kristensen and From, 2015b; Janssen and Verboord, 2015): ascribing value to products, thus setting “*a few reference points in this [music industry] jungle*” (C9) (**Judge**); legitimizing the cultural industry and acting as communication channel between artists and consumers (**Stakeholder**); and acting as creating agents who deliver valuable journalistic products (**Writer**). The role of **Stakeholder** was described by critics as central to their work, encapsulating the nature of criticism as the point of intersection between artists, industry and the public. In the words of critics: “…*the role of the review in the classical recording market is crucial*. *Without reviews the market would only half-function*, *because it needs to have the critical input*, *the validation from Critics*” [C2] and “*The role today of professional criticism? Well*, *it is that conduit from the producer to the public*. *It is that bridge*” [C4].

Beyond commercial interests, **Hats** revealed human-centered dimensions, in line with Cottle (2003) remark on how journalists harbor a genuine desire to serve the public. This is reflected in three further roles that critics described, which focus on the service offered to artists and consumers. Critics saw themselves as musicians’ **Advocates**, co-responsible to support the progress of an artist’s career, and at the same time as **Consumer Advisors**, pledged to provide guidance to purchasing and listening behavior. The words used by critics emphasize their feeling of responsibility toward both audience and artists, for example: “…*that’s the sort of thing I’m very*, *very aware of*. *I feel I’m doing it for the musicians*. *I’m writing for them*” [C2].

One last role that focuses the human-centered dimension of criticism is that of a **Teacher**. Critics saw in their work the potential to inform, illustrate, and educate, thus assisting listeners to understand and appreciate the music performance, but also offering feedback to musicians on the value of their artistic choices. As such, the music critic “*today is also a social critic*, *a teacher*, *a pedagogue*” with “*pedagogical duties to fulfil*” [C11]. The view of the critic as a teacher seems to partially reflect the early 20th century music appreciation movement (e.g., Scholes, 1928; Jorgensen, 1987; Prictor, 1998; Witts, 2011). It also resonates with Cone (1981) distinction between the role of the “reviewer,” whose aim is to guide listeners’ choices (here this would be the **Consumer Advisor Hat**) and the proper “critic,” whose aim is to broaden and deepen the reader’s appreciation of music. What seems unique to our critics’ description of their role, however, is that they see their pedagogical value addressed not only toward listeners, but also toward the musicians themselves.

One role that was largely absent from the interviews was that of “Journalist.” Our critics rarely used this term, preferring instead to define themselves as writers. This stands in contradiction to reports that arts journalists are increasingly seeking solidarity in news organizations as “reporters” (Hellman and Jaakkola, 2011). Our critics’ professional self-concept more closely follows an aesthetic paradigm that defines them as “connoisseurs and ultimately experts” (Barzun, 2001, p. 71) and “representative(s) of the artistic field in the newspapers” (Hellman and Jaakkola, 2011, p. 785). This may be a unique feature of classical music critics who have multiple opportunities to write specialized articles for general outlets, offering a critical product that is “less reportage than interpretation” (Muller, 2005, p. 105).

### Principles – Things I must be or have

In the second theme category, critics described eight core conventions or moral standards governing their writing. These principles find parallels in the five ideal-typical values of journalism proposed by Deuze (2007) and align with Harries and Wahl-Jorgensen (2007) who interviewed arts critics from a wider genre base and reported a set of rules that represents critics’ “code of conduct.”

Our critics principles align with the ‘Hats’ they described. Three principles revolve around critics’ main functions as assessors and stakeholders of the music industry: **Integrity**, **Authority**, and **Accepting Subjectivity** set out the grounds upon which critics’ judgments build and profile critics’ statements as, essentially, an informed opinion. Music critique judgments, according to our critics, should be based on a solid foundation of knowledge and extensive experience in the field, which give critics the **Authority** to command their position. At the same time, the critic should avoid normative statements and present him/herself as a provider of a well-informed, but ultimately personal judgment given at a particular time and place (**Accepting Subjectivity**). In a critic’s words: “*Because we do have this*, *kind of*, *idea*, *this false idea I think*, *that Reviewers are objective*. *I mean*, *you are objective to a certain extent*, *but a lot of it is based on subjective opinion*, *…you*, *kind of*, *have to accept it as part of it and say*, *well*, *this is an informed subjective opinion*, *but it’s still a subjective opinion*” [C7]. Critics’ call to accept the subjectivity inherent in any aesthetic judgment (Harries and Wahl-Jorgensen, 2007; Hellman and Jaakkola, 2011) counters the value of ‘objectivity’ in Deuze (2007) and reflects theories in aesthetics that date back to Hume’s Standard of Taste (Levinson, 2002; Budd, 2007) as well as current models in economics of information that set music as stereotypical “experience good” (Nelson, 1970; Mudambi and Schuff, 2010). According to our critics, objectivity in music criticism is replaced by expertise (**Authority**) combined with impartiality and truthfulness (**Integrity**). In particular, the **Integrity** principle in our critics’ words seems to them arise both values of “ethics” and “autonomy” found in Deuze (2007): Critics should remain true to their own response to music, free from prejudices or conflicts of interest, and open-minded to new ideas and interpretations. Critics define this as an “*element of courage in reviewing”* which requires people *“to stick their neck out”* and *“to be prepared to say what you believe*, *and what you think*” [4].

Building on these three pillars of critical judgments, two further principles focus on the human-centered dimension of critique, in line with critics’ roles as pedagogues, advocates, and consumers’ guides. In communicating their judgments, critics should be aware of and understand the expectations, efforts and standpoints of the people involved (**Respect**). Again, critics’ feeling of responsibility apply to both the audience and the artist, thus strongly resonating with Deuze (2007) dimension of “public service”: critics should have a keen sense of the audience’s knowledge and appreciate the readers’ perspective. At the same time, they should respect the musician’s feeling and sensitivity and actively try to understand what s/he may have tried to achieve. **Respect** toward the artist was described in interview as a fundamental rule of critique: “*The core principle is always …to take the person*, *who is offering me the recording*, *seriously*. *And this means that I have to ask myself*, *what does s/he want to tell me?*” [C11]. This in turn translates into a form of criticism which ought to be **Constructive**, to offer an evaluation that is potentially beneficial to the musician and avoiding a damning review: “*I do not like*, *basically*, *the negative criticism*. *I think criticism should …be constructive*. *You should be saying something which could be just possibly helpful*” [C8].

Building on the principle of constructing review, the last three principles described by critics focus on the way the review is written, setting critics’ writer role to the front. Interestingly, these principles reflect broadly Beardsley’s triadic theory of aesthetic value in the arts (Beardsley, 1962, 1982), which has been found to be reflected also in critics’ evaluations of music recordings (Alessandri et al., 2016a). In interviews, critics pledged for reviews to be immediately understandable to the reader, coherent and user-friendly (**Clarity**), to be engaging and pleasurable to read, able to catch the reader’s attention and arouse his/her curiosity (**Interesting**), and to represent and share the spirit of and passion for the music as well as a sense of the listening experience in words (**Capture**). Principles like **Capture**, **Interesting**, and **Clarity** accent a further dimension in music critics’ values and professional self-concept; as communicators, translators of knowledge, and sources of inspiration. The fact that these principles roughly align with the criteria of clarity, intensity and complexity that emerged in Alessandri’s analysis of published music reviews (2016a) emphasizes critics’ role as creative agents and suggests that music reviews – on top of the different functions they fulfill – might be seen as a work of art in its own right, as a piece of art evaluating art. Critics’ words in interview well convey the view of review as a creative product: reviews ought not just to be clear and informative, they have to ‘captivate’ and ‘charm’ the reader [C10] and even become the written essence of the music. As a critic said: “*I want to …endlessly recreate it in my work*, *to recreate a spirit of someone’s performance …in words*” [C8].

### Challenges – Things I feel about my job

After ‘Hats’ and ‘Principles’ the third theme category highlights six key **Challenges** that arise from the need to juggle responsibilities toward artists, audience, and the recording industry while remaining true to an implicit code of conduct. **Challenges** are discussed in terms of the circumstances that critics negotiate, and how this makes them feel.

Two **Challenges** highlight conflicts between critics’ roles, principles, and the context in which critics act. Critics are aware of the potential impact of their writing, both negative and positive (**Influence**). This is the challenge of having power, and critics discuss this in terms of potentially misleading consumers, damaging a person’s career or increasing/decreasing sales and publicity: “*I know that they are liable to use my words to advertise that CD and to advertise the Pianist in general*. *So*, *I’m aware of the power and the power of the press*” [C3]. The awareness of the impact of their critique, mixes with a feeling of **Pressure** arising from personal or indirect reports criticizing the critic’s work or encouraging them to provide a certain opinion or information in their review, or to use a certain tone or language.

The source of pressure can be the artist or their representatives but also the recording industry in general, including labels, magazines, record producers, and the dynamics between them. Conflicts of interests can arise from entertaining relationships with artists or having personal sympathies, and critics warn about getting “*too close to the people in the business*, *so close*, *you cannot be truthful*” [C2]. On the other hand, even in absence of sympathies or relationships, critics are aware of the possible consequences of their writing, in terms of reactions toward the critics themselves: *“…you have to always be thinking about the legal consequences*, *you do not want to libel anyone …you have to be quite careful with your language to make sure that you do not say anything that they could take you to court over*” [C7].

As stakeholders of the music industry critics feel pulled in different directions, stretched through the “*inextricable link*” between “*the commercial life of the record industry and …how record magazines cover these records*”: *“…although it would never say so*, *the Gramophone has an agenda*, *which is to promote current recording and the critical faculties will follow from that*” [C1].

Personal interests and biases as well as the consequences of their critique clash with the need for fair and impartial judgments (“**Integrity”** Principle) and the desire to guide and support consumers and artists (Hats “**Consumer Guide**” and “**Advocate**”). While conflicts of interest and pressure from the industry have been reported in other art criticism contexts (Chong, 2017), one challenge that does not find resonance in the literature is the feeling of responsibility that the music critics bear toward classical music artists, nurtured by the awareness of the impact of the press on musicians’ self-concept and career.

Besides **Pressure** and **Influence**, one further challenge points at an inner conflict between critics’ day-per-day job requirements and their creative and aesthetic needs (Hat “**Writer”**). In interview, critics bemoaned to some extent being asked to review the same music works many times or having limited freedom in the choice of what to review (**Limited Repertoire**): “…*if you want longevity with the magazines …you have to take what they send you*. *They tend to send you what you have done before*, *so there’s very little renewal in the Reviewer’s frame of reference*” [C1].

Finally, the last three challenges them arise the complexity of being a music critic in the context of the current communication and music consumption market: critics operate today in a large field of published opinion and coexist (and compete) with multiple novel channels of communication like blogs, Twitter or Amazon (**Market**): *“…the freeness of the Internet is a great boon in some ways*, *but it’s a disaster in others*, *because it’s overload*, *information overload …And*, *you know*, *it’s very difficult to weed out what opinions are worth reading*, *for readers*” [C4]. Their role as mediators in the music industry is more relevant than ever, and yet critics bemoaned this position in the modern consumer market “*Somewhere in-between a diffuse*, *heavily changing public*” [C12]. The complexity of this scenario raises questions on the very nature of the critic as consumers’ guide: “*I do not know how people are going to consume music*. *The question is if you have got YouTube and you have got iTunes and*, *you know*, *all these massive channels for acquiring music*, *how do you guide people and do people want to be guided?*” [C1]. This adds to a general feeling of disconnect that critics described, between the expected and actualized response to their work (**Recognition**). This includes issues around payment, misunderstanding of their aims or meaning, as well as a perceived overly negative portray of critics: “*And I’ve always said to people*, *‘Look*, *everybody hates Critics*. *Get your machine guns out’*” [C8]. These circumstances and the changes in the industry and consumer habits nurture in critics a deep concern for the legitimization and even meaningfulness of critical practice itself. In line with the recent acknowledgement of a ‘crisis’ within arts journalism (Jaakkola, 2015; Baía Reis, 2018), critics in interview shared thoughts about the **Uncertain Future** of their profession, including the idea that it is losing volume and significance. In the words of critics: *“…I think record criticism is declining*. *It will probably dissolve*” [C10] and *“…the social structures and social mores have given people confidence to make their own decisions*. *So*, *the role of the Critic’s not necessary*” [C1].

The first three theme categories above depicted critics’ role, the principles they follow, and the challenges they face. The lower half of the visual model features the two remaining themes of **Topics** and **Tools,** which focus on the strategies and devices critics employ to fulfil their purposes, be true to their principles, and deal with challenges.

### Topics – Things I discuss

**Topics** define the aspects of the recorded performance covered in review. This includes seven subthemes that detail comments on the context, the product, the music, and the critic’s reaction. The seven **Topics** that we identified are a good match to those reported previously, and which were based on the analysis of critical review content from one outlet, the Gramophone magazine (Alessandri et al., 2015, 2016b).

In line with those previous findings, critics in interview confirmed that the core content of their writing is the description and evaluation of the musical performance. This includes comments on style, originality, communication, interpretation, as well as comments on musical parameters like tempo or phrasing (**Performance Achievement**). For example: *“…you are saying how the performance is*, *what was good about the performance*, *you know*, *the expression and the phrasing*” [C4] or *“…these are important things to cover*. *The liveliness of the Gestaltung and of course the faithfulness to the text*” [C9]. As can be seen from these excerpts, in the interviews critics did not go into detail on the discussion of the **Performance Achievement**, limiting themselves instead to a few examples of themes therein. When asked for more details they tended to provide concrete musical examples, instead of venturing into a general categorization of performance. This pattern of behavior makes sense when interpreted in the light of previous findings; discussion of a performance tends to form the largest part of review and is characterized by a complex variety of descriptive and value-laden constructs.

The discussion of the performance merges in review with the description and evaluation of the recorded sound (**Recording Quality**), consistent with Alessandri et al. (2016b). In line with Philip (2004) and Patmore and Clarke (2007) critics thematized within this theme the importance of recreating through the recording the impression of a live performance, “*I will certainly comment on* …*whether it sounds like a studio recording or whether the artist had been able to transcend the recording studio and give me the impression that it’s a live important event*, *which is just happening*” [C3].

In addition to the performance and the recorded sound the recording product itself is an object of discussion in review (**Package**). This topic clusters different sub-level themes found in Alessandri et al. (2016b), which are all elements *extra to the actual sound*, e.g., the program performed, the composer, the instrument and score edition used, but also sleeve notes, cover art design, comments by the artist or issues of translation.

Two further **Topics** are used to contextualize the recording in terms of its history and its **Place** in the emerging market, and to offer information about the performing **Artist**, their career, school their come from, track record of recordings and general skills: “*I always like to contextualize a record*, *you know*, *when was it made? Why was it made? Who was it made for? What were the circumstances around the recording?*” [C1]; *“…I would then offer a few background information*. *Biographical information of the interpreter*, *what has s/he done so far*, *to introduce the musician a bit*” [C12].

The last two **Topics** focus on the critic’s affective reaction to the music (**Response**) and his/her recommendation for the reader, in terms of whether to buy or whether and how to engage with the recording (**Advice**). These last **Topics** are the only ones that do not find a direct correspondent in the music review model from our previous work (Alessandri et al., 2015, 2016b). This discrepancy might be explained by the fact that critic’s **Response** and **Advice** are always about an element of the recording – e.g., the artist, the performance, or the recorded sound – and thus have been coded in the previous work under such themes. That critics in interviews described these as separate categories offers new insights concerning the motivation behind those statements, which strongly aligns with critics’ felt responsibilities as advisors and teachers (Hats). The weight given to the critic’s own **Response** to music also resonates with the typical amount of affective evaluative terms (e.g., moving; daunting; cloying) used in reviews (Alessandri et al., 2015) and with findings on the importance of emotional response for the evaluation of art (Chong, 2017).

The high convergence of evidence between what critics told us about their writing and what emerged previously from the text analysis of published reviews adds support to the proposed model structure and construct conceptualization. Moreover, this indicates that the model previously developed from only one specialized magazine reflects well the content from other sources and authors. One area of music critical writing untouched by previous examination of reviews were the writing strategies employed to discuss and structure topics; these would have been impossible to presume from the written source alone. These writing strategies are the focus of the last theme category.

### Tools – Things I use when writing

This theme category entails nine subthemes containing comments on devices and strategies that critics may employ whilst reviewing a recording, i.e., from the minute they listen to the music to the final production of the document.

The first two **Tools** concern the act of **Listening** itself and the reliance on colleagues, editor, and/or the artist(s) **Feedback** during the review process. Critics in interview emphasized the importance of using a high-quality reproduction system (at least at some stage of listening) and having the score to hand as reference material while listening. They also reported that they actively seek discussion with colleagues or artists to clarify questions or just have “*an informed discussion*” about the recording [C7]: “*It also happens that I actually call the agency or the CD label and say: ‘I would like to briefly talk to the pianist*. *I simply have specific questions*.*’ …then we talk*” [C11].

A further block of themes within **Tools** covers structural and literacy devices used in writing. In line with previous findings (Alessandri et al., 2015) and with anecdotical reports (Pollard, 1998) **Comparisons** between the recording reviewed and other recordings or related experiences (e.g., seeing the performance live) are a common device in critics’ writing toolbox: “*A bad review considers the work or the CD as an individual object*. *…But a good one shows that this CD indeed does not stand alone*, *it is anchored in a wider space*, *in a repertoire*” [C14]. In addition, the use of a concise writing style was presented as an essential requirement in review practice and in journalism in general (**Brevity**): *“…space is the crucial thing*, *always*, *with journalism*. *…if you are only allowed a small slot*, *you must keep within that slot and it does limit what you can do*” [C2]. In their study of arts journalism in Finland, Hellman and Jaakkola (2011) interpreted a reduction in review length as a sign of a shift from the aesthetic to the journalistic paradigm. Indeed, all our critics discussed word counts as a challenge to a thorough and insightful music review. However, they framed this issue in terms of the need for concise writing skills and awareness of target audience rather than as an invitation to change their approach. This concise writing style was reflected in the high density of themes per clause found in previous analysis of written text (Alessandri et al., 2015).

A third structural writing tool concerns the use of story elements within the review, like a clear headline, distinct opening or closing statements, and a core message or angle. In reviews critics offered concrete examples of their preferred **Narrative Structure**: *“…begin with a fanfare*. *So get the reader’s attention*. *And end with a cadence*, *so you get the feeling at the end that*, *yes*, *this is the end of the review*. *We have come to a conclusion*” [C6].

Critics also commented on the use of different vocabulary and linguistic styles to describe the recording (**Language**), for instance weighing the use of musical terms (e.g., fermata, counterpoint, Leitmotiv; **Specialized**) and figurative speech (like metaphors, similes or personifications; **Symbolic**) according to the target audience, or using wit, satire or irony (**Humor**) as well as first and third person (**Distance**) to shape the character of their writing. In line with the Principles of **Respect** and **Clarity**, critics found that music specialized terms should be used sparely, while metaphors and similes were appreciated as a way to “*color your writing*” [C5]. Mixed feelings were expressed toward the use of quantified evaluations in review, like numbers or stars (**Rating**). Some critics found these could be an added value for consumers, facilitating comparison between recordings, while other warned about the risk of over-reducing the critical appreciation to a quantified value: “*The star system simplifies things at times*, *but this simplification takes away the possibility to undergo very differentiated experiences*” [C12].

The last two **Tools** that the critics discussed appeal to more abstract but essential strategies in reviewing: the accuracy and diligence in the reporting of details about a recording (**Thoroughness**) and the justification of value judgments by means of factual and rational writing combined with the use of examples to back up assessments (**Justify**). The act of adducing reasons to support evaluative judgments was described by critics as the fundamental nature of reviewing, “*the essence of all of this is about …you have to justify and make clear your process of thinking*” [C5].

The importance of grounding critical judgments of recorded music in reason resonates with a wider philosophical debate on the nature of criticism, which sees the reasoning process behind evaluation as the defining trait of critique, as opposed to a more information and description-based journalism (Cone, 1981; Beardsley, 1982; Carrier, 1986; Davies, 2001; Danto, 2002; Carroll, 2009). This understanding is also grounded in the history of the critical practice (Carroll, 2009, p. 16). We find this assumption for instance already underpinning the two seminal essays on music criticism by Calvocoressi (1923) and Newman (1925), and also, 40 years later, in Walker’s *An Anatomy of Musical Criticism*, in which this idea is stated explicitly at the opening (1968, p. xi):

“The practice of criticism boils down to one thing: making value judgments. The theory of criticism, therefore, boils down to one thing also: explaining them. If you formulate a theory of criticism, it is not enough to know that one work is a masterpiece and another is a mediocrity. You must also explain why they are different.”

The previous work done by the authors (Alessandri et al., 2015) supported this view based on a large sample of evidence from published reviews. The analysis showed that critics’ texts contained a large variety of descriptors adduced as reasons to support judgments. Descriptors were divided into two major categories, which resonate with the different use of **Specialized** and **Symbolic** language: technical constructs like sound parameters and mechanics of delivery, and abstract constructs like character, structure, or style, where critics made use of metaphors and similes to convey their impressions.

The current interview work further adds to these previous findings, confirming that this very quality of review is intentional and that critics are well aware of this. In the words of one of our critics: “*You argue*. *You reason*, *exemplify*, *and justify*. *This is critique*” [C11].

The emphasis on reasoning given by our music critics thus supports a professional self-concept distinct from that of a more general journalist or reporter. The use of rhetorical and stylistic tools as first-person or wit, together with assertions on the importance of emotional **Response**, is in line with literature describing the increasing relevance of emotion-related statements in music criticism as a means to achieving a more engaging and direct form of communication (Wahl-Jorgensen, 2012; Coward, 2013).

Taken together, the **Tools** category again offers a good match with the results of previous analysis (Alessandri et al., 2015, 2016a,b), noting that this has been the first opportunity to verify the contents of written review with verbal confirmation of the intention behind the source output.

## Discussion

We interviewed 14 expert music critics from United Kingdom, Germany, and Switzerland to understand how they view their role and practice. The resultant visual model offers a detailed, reflective map of the nature of criticism in the classical music market, comprising music critics’ opinions and beliefs regarding their impact on consumers and artists as well as how these thoughts inform their writing process.

### Critics in the modern classical market

The model generated from interviews with critics self-identifies them as “cultural intermediaries” (Bourdieu, 1984, p. 325) between classical music producers, artists and consumers: As a bridge that fulfils a variety of purposes for each industry stakeholder.

Following Kristensen and From’s (2015b) typology, our music critics can be included under the heading of cultural journalists: passionate professionals, who aim to deliver aesthetic evaluations grounded in clear reason (*Judge*; *Justify*) while offering an engaging literary product (*Writer*; *Interesting*). Their profile defines them as intellectual cultured critics, driven by a sense of responsibility to create accessible and relatable knowledge for all their perceived stakeholders (*Teacher*, *Clarity*, *Respect*; *Constructive*). Our critics remain “devoted to the comparison and analysis, to the interpretation and evaluation” (Cuddon, 1982, p. 207), triggered by the feeling of “fulfilment of a duty toward a matter” (Adorno, 1998, p. 142).

The music critics voiced beliefs in line with those of other arts journalists, highlighting their role beyond the news agenda (Harries and Wahl-Jorgensen, 2007). In line with Kristensen and From’s (2015b) typology and Harries and Wahl-Jorgensen’s (2007) theory of “arts exceptionalism,” our findings clarify the cultural journalist profile and show how it is experienced by seasoned music critics both in terms of responsibility and concerns. In positioning themselves squarely in the aesthetic paradigm of occupational professionalism (Örnebring, 2009), many music critics report struggling with an arts journalism archetype that is shifting toward a media-led organizational standard.

Our critics were aware of their multiple roles within the music market and the potential for controversial consequences (*Stakeholder*; *Consumer Advisor*; *Pressure*; *Influence*; *Market*), and yet they emphasized their drive to be conveyors of culture, advocates of music and of musicians, and teachers (*Teacher*; *Advocate*; *Respect*; *Constructive*; *Capture*; *Interesting*). In a music world that often appears to be dominated by prejudices against them (Brennan, 2006), the critics’ pledge to the aesthetic paradigm: their passion for music combined with their desire to share knowledge and serve musicians and listeners alike emerged from the interviews as a call for understanding and acknowledgment (*Recognition*).

### Is professional music criticism dying?

The call for recognition amongst music critics gains urgency in the context of new opinion sharing and communication channels. Our music critics identified online blogs and digital magazines as both a resource and a threat to quality criticism (*Market*). This conflict reflects a wider debate on the shifting role of journalism in the digital era (Agarwal and Barthel, 2015). Our music critics observe this shift with concern and scepticism; in line with Deuze (2007), they fear a marginalization of professional critique in the digital media age (*Market*; *Uncertain Future*). However, the opposite position in this debate, that of a constructive integration of professional criticism into a hybrid media system (Chadwick, 2013) was also reflected. Some critics entertained the idea of fusing traditional and new practices to redefine the nature of music critique in the near future.

Of deeper concern to our participants was the perceived *raison d’être* of music criticism in view of modern music consumption. In the age of Spotify and YouTube, the critics questioned what kind of guidance, if any, consumers need when music is low cost (or free) and selected by computer algorithms. This question was accompanied by feelings of resignation and marginalization (*Recognition*, *Uncertain Future*), but was also met by a strong sense of purpose and self-identity: classical music critics emphasized the importance of their autonomy, today more than ever. They outlined a set of norms (**Principles**) and job roles (**Hats**), grounding their critical identity firmly in their unique expertise (*Authority*), aesthetic purpose (*Teacher*), and third-party perspective (*Judge*; *Integrity*; *Comparison*).

### Informed judgment as added value

Critics described their ultimate value in terms of a benefaction for the music listening public. Their skill is in taking the aesthetic response that we all experience and transforming it into a public discourse. Only by this transformation does the aesthetic judgment obtain importance: “Through the relationship with the reading public, critical reflection loses its private character.” (Eagleton, 1984).

The justification of aesthetic judgments in terms of *Authority* and *Respect* seems at first glance to reflect an elitist image of the cultural critic, which conveys not only knowledge but also actions to consumers (Dahlgren, 2012). However, music critics are clear that what they offer is only an informed evaluation (*Accept Subjectivity*). Its value resides in their knowledge as well as the principles and journalistic skills embedded in and sparked by – paraphrasing one of the critics – a burning desire to share their lifelong love for music.

In a consumption market characterized by quick and free opinion, stars and thumbs up, classical music critics pleas for deeper engagement with their text and with music listening are challenging. However, the market is expanding in terms of music devices and recordings (Krause et al., 2013) and one immediate consequence of this trend is paralysis of choice (Schwartz, 2008); consumers can be left unsatisfied with the music selected for them and feel mislead by judgments that they perceive as ill-informed or created by artificial means. This situation has led to some advocating random selection of music as the only reasoned approach (Leong et al., 2008). It is an irony that the same market which critics have come to view with suspicion may need them now more than ever.

### The job of music critique

The act of music criticism has been defined as “the translation and grading of an aesthetic experience by means of intellectual analysis and imaginative inquiry” (Dean, 1980, p. 44). By asking critics about their practice, we gained insights into the tools of this trade that enable them to produce quality content.

The subheadings within **Topics** and **Tools** provided a good match with the constructs from previous written review analysis (Alessandri et al., 2015, 2016a,b), indicating a high correlation between intent and outcome in critical review. However, within the current model, aspects of recordings and the way these are discussed did not play a central role. Rather, **Hats**, **Principles,** and **Challenges** emerged as dominant, complex theme clusters. This meta-reflection on the job of being a music critic usually remains hidden to the reader. In previous analyses, it was shown that critics can sporadically let “slip” their thoughts about review writing, its processes and challenges (*Meta-Criticism*, Alessandri et al., 2016b). However, this was always a minor point, and it is interesting to note that none of the critics in the present paper mentioned that they wrote about these issues. This suggests that sporadic meta-reflections on the critical practice itself in review reflect an inner need for explanation and understanding.

## Conclusion

Critical reviews of music recordings are a common and relevant form of performance evaluation. Building on previous *post hoc* research on the content of critical writing, in this article we report findings from interviews with professional critics that offer insights on the intentions, motivations, and principles behind this well-established form of critical appraisal. Our visual model of music critique brings together many layers and facets of critics’ professional self-concept in combination with their experience and practice. As such, it adds a new dimension to the music criticism literature and gives insights into the mechanisms and reasons behind critics’ evaluations and into the key elements of critical reviews, which experts see as influential and relevant for consumers. It also shows the challenges critics face, standing as they do in an intermediate position between the producers and consumers of classical music, as well as straddling a complex intersection between artist, journalist and educator.

Ultimately, these findings offer a new interpretative viewpoint on critics’ aesthetic judgments and on their perceived place within the digital classical musical world. They bring a message of hope: Although many critics spoke of their fear for the future, the engaged and multifaceted evaluative approach they bring to music gives good reason to believe that their unique abilities will be in increasing demand by the sophisticated music consumer who asks for more and not less informed control over their choices.

## Data availability statement

The datasets presented in this article are not readily available because the sharing of the original interview transcripts would mar the anonymity requirement. Relevant excerpts from interviews are included in the manuscript and in the supplementary material. Requests to access the datasets should be directed to the corresponding author.

## Ethics statement

The studies involving human participants were reviewed and approved by Research Ethics Committee, Department of Music, University of Sheffield. The patients/participants provided their written informed consent to participate in this study.

## Author contributions

VW was responsible for the ethical approval. EA ran the interviews. EA and VW wrote the first draft of the manuscript. All authors contributed to the design of study, participant recruitment, and data analysis, reviewed and edited the manuscript, and approved the final version.

## Funding

This study was supported by the Swiss National Science Foundation (grant number 100016M_162819). Open access funding provided by Lucerne University of Applied Sciences and Arts.

## Conflict of interest

The authors declare that the submitted work was carried out in the absence of any personal, professional, or financial relationships that could potentially be construed as a conflict of interest.

## Publisher’s note

All claims expressed in this article are solely those of the authors and do not necessarily represent those of their affiliated organizations, or those of the publisher, the editors and the reviewers. Any product that may be evaluated in this article, or claim that may be made by its manufacturer, is not guaranteed or endorsed by the publisher.
