# Supplementary material for: The critic’s voice: On the role and function of criticism of classical music recordings
Source: Front Psychol. 2022 Sep 29;13:925394. doi: 10.3389/fpsyg.2022.925394 (PMC9557232; doi:10.3389/fpsyg.2022.925394)
Supplement: Supplementary file 1 [file Table_1.docx]

## The Critic’s Voice Alessandri, Baldassarre & Williamson

## Supplementary Material 1 – The Critics

**List of interviewees**

| *Critic* | *Years of Experience* | *Major Activity Venues as Reviewer* |
| --- | --- | --- |
| C1 | 37 | Record Collector; Gramophone; Sounds; Fugue; Journal of the Association of Recorded Sound Collections; Classical Recordings Quarterly |
| C2 | 25 | The Times; The Independent |
| C3 | 24 | Gramophone; International Piano; Classic FM Magazine; Classic CD; BBC Music Magazine; International Record Review |
| C4 | 30 | London Evening Standard; The Times; BBC Music Magazine; The Tablet; The Guardian; The Independent; Daily Mail; Sunday Times; Classical Music; Washington Post |
| C5 | 25 | Gramophone; International Records Review; Piano Quarterly |
| C6 | 47 | Birmingham Post |
| C7 | 50 | Gramophone; BBC “Building a Library”; ABC; CBC; Grove’s Dictionary of Music and Musicians |
| C8 | 5 | BBC Music Magazine; The Times; International Piano; Shakespeare Magazine |
| C9 | 39 | Tages-Anzeiger Zürich; Radio DRS2; Die Wochenzeitung |
| C10 | 40 | Basler Volksblatt; Basler Nachrichten; Frankfurter Allgemeine Zeitung; Hessischer Rundfunk; Neue Zeitschrift für Musik |
| C11 | 20 | Fono Forum; Neue Musikzeitung; Neue Zürcher Zeitung; Die Welt; Abendzeitung; Musik & Theater; Das Orchester; Piano News |
| C12 | 32 | Fono Forum; Rondo |
| C13 | 25 | Fono Forum |
| C14 | 25 | Fono Forum; Die Zeit; Klassik Heute; Fanfare; Frankfurter Allgemeine Zeitung; Phonographic Bulletin |
