# Supplementary material for: The critic’s voice: On the role and function of criticism of classical music recordings
Source: Front Psychol. 2022 Sep 29;13:925394. doi: 10.3389/fpsyg.2022.925394 (PMC9557232; doi:10.3389/fpsyg.2022.925394)
Supplement: Supplementary file 2 [file Table_2.docx]

## The Critic’s Voice Alessandri, Baldassarre & Williamson

## Supplementary Material 2 – The Interview

**Interview schedule – English**

- Prompts are given in *italics*: These are not questions but rather ideas to extend the conversation if responses are short or the person requests clarification.

Intro

Thank you for being willing to take part in this interview. First, let me assure you that you will remain completely anonymous and that we will not keep any identifiable records of the interview.

I am Research Fellow at the [Name of University], and with [Name of Researcher] from the [Name of University] we are researching the role of professional review of recorded performance in the classical music market.

[Paragraph omitted to preserve anonymity].

In this interview I am really interested to hear about your opinions and personal experiences – this will help us improve understanding of the nature, purpose, and influence of recording critical review. You are more than welcome to interrupt, ask for clarification, criticise a line of questioning, and so on.

Finally, before we start – have I got your permission to record this interview? I can then listen to what you are saying instead of having to write everything down, though I will make the occasional note as we talk.

Our conversation today focuses on reviews of recordings of classical piano repertoire. There are three main points I would like to address:

1. **what** are the **aspects** of a recorded performance you cover in your reviews?
2. **how** do your write about these aspects in terms of stylistic and rhetorical devices as well as positive and negative evaluations, and
3. **what** is in your opinion the **role** of professional criticism in our classical recording market?

First, just to get our conversation started, I would like to ask you:

Opening

1. Can you tell me about a recent recording of classical piano repertoire that you enjoyed?

Review Content

In the first part of the interview, I would like to talk with you about **what** you write about when reviewing a recorded performance of classical piano repertoire. In doing so please think of the kind of critical review you most often write.

1. With this review format in mind, I would like to first ask you about the **content** of what you write as opposed to your comments on the performance itself. I intend to focus on those comments in a minute. Could you tell me, in no particular order, what are the most important **elements** to cover when reviewing a **recorded performance**, other than the comments on the performance itself?^[[1]](#footnote-1)^

*Is there a formula or typical map you use to structure the discussion of these elements?*

*To what extent do you have free choice in deciding what elements should be discussed and to what extent do you have to follow editorial instructions?*

*How important is it to compare the recording reviewed with other recordings?*

1. Now that you mapped out the different elements of a recording review, I would like to focus on **how** you write about **performance** itself. Could you tell me in your opinion what are the most important **aspects** **of performance** to cover?

*Is there a formula or typical map you use to structure the discussion of these elements?*

*How important is it to compare the recording reviewed with other recordings?*

*What is the role of metaphor and other rhetorical devices in your writing?*

*What role plays technical language in your writing?*

*To what extent do you have to follow editorial instructions concerning the use of rhetorical devices, metaphors, and technical language?*

*Tell me about your usage of positive and negative statements in your critical writing*

Review Function

Thank you very much. In the next part of the interview I would like to change our subject to focus on the function of reviews.

1. We have been talking about the kind of review you most often write. What are the main things that change when you write for **different audiences**?

*Are there differences in the choice of aspects to discuss?*

*Are there differences in formula or typical map you use to structure your review?*

*Are there differences in the use of metaphors and rhetorical devices?*

*Are there differences in the use of technical language?*

1. What do you consider to be the **purpose** of your critical writing?
2. Based on your experience with the current music market, what do you think is the **role** of the professional critical review in our classical recording market?
3. Finally, what in your opinion is the **influence** of professional music criticism on the reader as opposed to standard forms of peer review that are widely available, like those you find on Amazon?

**Interview schedule – German**

- Anregungen/Aufforderungen werden in *Kursivschrift* dargestellt: Dabei handelt es sich nicht um Fragen, sondern Ideen, um das Gespräch zu erweitern, wenn die Antworten kurz ausfallen, bzw. um vom der befragten Person gewünschte Erläuterungen.

Einleitung

Vielen Dank für Ihre Bereitschaft, an diesem Interview teilzunehmen. Zuerst möchte ich Ihnen versichern, dass sämtliche Daten anonym behandelt (werden) und wir keine identifizierbaren Daten des Interviews aufbewahren werden.

Ich bin wissenschaftliche Mitarbeiterin am [Name of University]. In Zusammenarbeit mit Frau [Name of Researcher] der [Name of University] führe ich ein Forschungsprojekt durch, in welchem die Rolle von professionellen Kritikern im E-Musikmarkt untersucht wird.

[Paragraph omitted to preserve anonymity].

Ich bin also sehr an Ihren eigenen Erfahrungen und Ihrer Meinung als Experte/in interessiert – das wird uns helfen, ein besseres Verständnis über das Wesen, den Zweck und den Einfluss von Kritiken über Tonaufnahmen zu erhalten. Bitte zögern Sie nicht, mich zu unterbrechen, falls Ihnen etwas nicht klar sein sollte oder Sie die Art und Weise der Befragung kritisieren möchten.

Bevor wir mit dem Interview beginnen, möchte ich Sie noch fragen, ob Sie einverstanden sind, dass wir das Gespräch aufzeichnen: so muss ich während des Interviews nicht alles niederschreiben, werde mir aber – wenn nötig – Notizen machen.

In unserem Gespräch geht es um die Kritik von Aufnahmen aus dem Klavierrepertoire. Ich möchte mit Ihnen auf drei Themen eingehen:

1. **Welches** sind die **Elemente** einer Aufnahme, die Sie bei Ihrer Kritik beachten?
2. **Wie** bringen Sie diese Elemente **sprachlich** und **stilistisch** sowie mit Blick auf positive und negative Urteile zum Ausdruck?
3. **Welche** **Funktion** hat Ihrer Meinung nach die professionelle Musikkritik im Tonträgermarkt?

Darf ich Sie nun als erstes fragen:

Einstieg

1. Können Sie mir etwas über eine aktuelle Aufnahme sagen, welche Ihnen gefallen hat?

Inhalt der Kritik

Nun möchte ich mit Ihnen gerne darüber sprechen, **worüber** Sie schreiben, wenn Sie eine **Tonaufnahme** aus dem klassischen Klavierrepertoire beurteilen. Bitte denken Sie an das von Ihnen am häufigsten verwendete Kritikformat.

1. Davon ausgehend möchte ich Sie zuerst zum **Inhalt** der Kritik befragen, der sich nicht auf die Ausführung bzw. Interpretation bezieht. Auf Interpretationsurteile werden wir gleich zurückkommen. Können Sie mir also – ohne bestimmte Reihenfolge – sagen, welches für Sie die **Aspekte einer Aufnahme** sind, die Sie beim Verfassen einer Kritik beachten?

*Wenden Sie für die Darstellung dieser Aspekte ein bewährtes Muster an?*

*Können Sie frei darüber entscheiden, welche Aspekte besprochen werden müssen, bzw. müssen Sie redaktionelle Vorgaben befolgen?*

*Wie wichtig sind Vergleiche mit anderen Aufnahmen?*

1. Nachdem wir nun über die Aspekte einer Aufnahme gesprochen haben, möchte ich mich auf die **musikalische Ausführung bzw. Interpretation** konzentrieren. Können Sie mir bitte sagen, welche **Elemente** Sie besonders beachten, wenn Sie über die Ausführung und Interpretation schreiben?

*Wenden Sie für die Darstellung dieser Aspekte ein bewährtes Muster an?*

*Wie wichtig sind dabei Vergleiche mit anderen Aufnahmen?*

*Welche Rolle spielen Metaphern und andere Stilmittel in Ihren Kritiken?*

*Welche Rolle spielt die Fachsprache in Ihren Kritiken?*

*Macht die Redaktion Vorgaben in Bezug auf die Verwendung von Stilmitteln, Metaphern und Fachsprache?*

*Können Sie mir sagen, wie Sie positive und negative Äusserungen in Ihrer Kritik einsetzen?*

Funktion der Kritik

Vielen Dank! Nun möchte ich den Fokus unseres Gespräches auf die Funktion von Kritiken legen.

1. Wir haben uns bis jetzt auf das von Ihnen am häufigsten verwendete Kritikformat konzentriert. Gibt es Unterschiede, wenn Sie **für die eine oder andere Leserschaft** schreiben?

*Gibt es Unterschiede bei der Auswahl an Aspekten, welche besprochen werden sollen?*

*Gibt es Unterschiede hinsichtlich der von Ihnen verwendeten Muster für die Strukturierung der Kritik?*

*Gibt es Unterschiede bei der Verwendung von Metaphern oder anderen Stilmitteln?*

*Gibt es Unterschiede bezüglich der Verwendung der Fachsprache?*

1. Welche **Absicht** verfolgen Sie mit Ihrer Kritik?
2. Welche **Rolle** hat für Sie eine professionelle Kritik im Kontext des Tonträgermarkts, wenn Sie an Ihre eigene Erfahrung mit dem aktuellen Musikmarkt denken?
3. Abschliessend möchte ich Sie noch fragen: Welchen **Einfluss** hat Ihrer Meinung nach die professionelle Musikkritik auf die Leserin bzw. den Leser im Gegensatz zu den weitverbreiteten Formen der nicht-professionellen Beurteilung, wie man sie zum Beispiel bei Amazon findet?

1. If: ‚It depends‘. Then: I’m aware that review content, style, and structure may vary greatly depending on publication venue and recording reviewed. What I’d like to try is to extrapolate from this variety an ‘average review’ [↑](#footnote-ref-1)
