# Supplementary material for: The critic’s voice: On the role and function of criticism of classical music recordings
Source: Front Psychol. 2022 Sep 29;13:925394. doi: 10.3389/fpsyg.2022.925394 (PMC9557232; doi:10.3389/fpsyg.2022.925394)
Supplement: Supplementary file 3 [file Table_3.docx]

## The Critic’s Voice Alessandri, Baldassarre & Williamson

## Supplementary Material 1 – The Critics

**List of codes emerged from the analysis together with relevant definitions.** Codes are clustered in theme families, for each lower-level code one or more examples from the interview text are given.

| **Hats – Things I am** | | |
| --- | --- | --- |
| Advocate | To give publicity and support the progress of an artist’s career. | *“…the power of the press is considerable, in terms of the effect it can have on the career of musicians …that’s the sort of thing I’m very, very aware of. I feel I’m doing it for the musicians. I’m writing for them”* [C2] |
| Stakeholder | To legitimise elements within both the recording and the publishing industry and to act as a communication channel between them and the consumers. | *“…the role of the review in the classical recording market is crucial. Without reviews the market would only half-function, because it needs to have the critical input, the validation from Critics is important”* [C2]  *“The role today of professional criticism? Well, it is that conduit from the producer to the public. It is that bridge”* [C4] |
| Consumer Adviser | To provide listeners with guidance on purchasing and listening behaviour. This includes providing information on the existence of given products as well as encouraging readers to engage with music. | *“…you’re actually writing for the buying public. …You’re actually writing, I think, first and foremost, to say, is this a record worth buying or not?”* [C5]  *“That’s a good Critic doing his job, isn’t it, persuading somebody to listen to something he would never thought of listening to otherwise?”* [C3] |
| Teacher | General comments on showing, illustrating, or informing. |  |
| *Audience* | The above definition of teacher, to assist the audience to understand or appreciate the recording. | *“A Critic today is also a social critic …a teacher …a pedagogue. One has pedagogical duties to fulfil”* [C11] |
| *Artist* | The same definition of teacher, to offer musicians feedback on the value of their performance and their artistic choices. | *“…the first people to read a review are the performers you’ve written about. …They want to know that somebody’s been there and appreciated what they’ve done. Or not appreciated it. But the not appreciating is also of value to them, if it’s done right”* [C4] |
| Judge | To act as the assessor for the reader, to provide a verdict. Judgement also involved assessing whether the recording performance has reached pre-set personal or industry standards/whether it is even worthy of review. | *“…my job, as a Critic, is to play a part in that industry by …promoting people and performances, which I think are good and by not with ones that are bad”* [C2]  *“…this could be a function of professional criticism …that you set a few reference points in this jungle, to which people can relate to”* [C9] |
| Writer | To produce a valuable piece of writing, to be read by people. It includes comments on the skills needed to write music critique. | *“I think a review ought to be a nice little piece of literature, which is worth keeping”* [C6] |
| **Principles – Things I must be or have** | | |
| Integrity | Impartiality in the evaluation of recordings. It includes comments on being truthful to your own response to the music, free from prejudices or conflicts of interest, and being open-minded to new ideas and interpretations. | *“It’s a brave thing of people to stick their neck out and say, you know, that you are doing it wrong. So, there is an element of courage in reviewing. It sounds rather grand, but you have to be prepared to say what you believe, and what you think”* [C4] |
| Respect | To be aware of and understand the expectations, efforts and standpoints of the people involved. |  |
| *Artist* | The critic should respect the artist’s feelings and standpoint as well as the work gone into the production. It includes comments on using empathy and sensitivity to actively try to understand what the artist may have tried to achieve. | *“The core principle is always …to take the person, who is offering me the recording, seriously. And this means that I have to ask myself, what does s/he want to tell me?”* [C11] |
| *Audience* | The critic should have a keen sense of the audience’s knowledge, training, and understanding. Understand and respect the readers’ perspective, expectations and knowledge. | *“…you must not assume the person who is reading this …has heard of Chopin. You mustn’t assume that. I mean, most people reading Gramophone, of course they will. But you can’t …just necessarily assume that you’re talking to, always to a cultured, educated, sophisticated person who knows about music. I write for people”* [C3] |
| Authority | To be an expert on the subject and to have the level of extensive or specialized knowledge to command that position. | *“You can only say certain things if you have experience …somebody might write a sentence which can only have come from years of experience. From years of listening”* [C4] |
| Interesting | To be engaging and pleasurable to read, to catch the reader’s attention and arouse his/her curiosity. | *“…you’re really throwing a pebble into a pond. You try to say something which sends out a few ripples so the people reading they think oh, yes, yes. Oh, I must listen to that”* [C8]  *“You have to win your reader with your text. You have to charm him! You have to captivate him”* [C10] |
| Clarity | To be immediately understandable and explicit to the reader, coherent and user-friendly. Conversely, this also refers to the need not to oversimplify in the aim of clarity. | *“Have I said it in the simplest way, the clearest way possible? Have I used a long word where I can use a short word? And that’s not to …say that you can’t use complex language, you can, but it has to be …immediately understandable and immediately clear to the reader”* [C7] |
| Constructive | To offer an evaluation that is potentially helpful for the musician. Here critics comment on the need to focus on the positive aspects of the recorded performance or refuse to review a recording altogether to avoid a damning review. | *“I don’t like, basically, the negative criticism. I think criticism should …be constructive. You should be saying something which could be just possibly helpful”* [C8] |
| Accepting Subjectivity | The critic should present him/herself as a provider of a well-informed but ultimately personal opinion given at a particular time and place. | *“Because we do have this, kind of, idea, this false idea I think, that Reviewers are objective. I mean, you are objective to a certain extent, but a lot of it is based on subjective opinion, and I don’t think there’s any way of getting away from that, or – and I don’t think we should get away from that really. …you, kind of, have to accept it as part of it and say, well, this is an informed subjective opinion, but it’s still a subjective opinion”* [C7] |
| Capture | To represent and share the spirit of and passion for the music as well as a sense of the listening experience in words. This also includes comments on the difficulty to use words to describe and represent music. | *“I want to feel – or endlessly recreate it in my work, to recreate a spirit of someone’s performance on – in words, as far as I can”* [C8] |
| **Challenges – Things I feel about my job** | | |
| Influence | The potential impact of review, both negative and positive. This is the challenge of having power, especially discussed in terms of potentially misleading consumers, damaging a person’s career or wellbeing or increasing sales and publicity. | *“There is a certain fear …to offend or to hurt someone”* [C10]  *“I know that they are liable to use my words to advertise that CD and to advertise the Pianist in general. So, I’m aware of the power and the power of the press”* [C3] |
| Pressure | Personal or indirect reports criticizing the critic’s work or encouraging them to provide a certain opinion or information in their review or to use a certain tone or language. Here critics also commented on conflicts of interest linked to personal sympathies or relationships. |  |
| *Artist* | The source of the pressure is the artist or their representatives. | *“The danger for Critics is to get too close to people in the business, so close you can’t be truthful”* [C2]  *“…you have to always be thinking about the legal consequences, you don’t want to libel anyone …you have to be quite careful with your language to make sure that you don’t say anything that they could take you to court over”* [C7] |
| *Industry* | The source of the pressure originates from the recording industry, including labels, magazines, record producers, and the dynamics between them. | *“…the commercial life of the record industry and what and how record magazines cover these records, is inextricably linked. …although it would never say so, the Gramophone has an agenda, which is to promote current recording and the critical faculties will follow from that”* [C1]  *“Meanwhile, it goes so far that the labels exert pressure as to not publish any damning review; and no damning reviews are published anymore”* [C12] |
| Limited Repertoire | The critic bemoans to some extent being asked to review the same music works many times or having limited freedom in the choice of what to review. Comments on the difficulties of finding something new to listen for, identify, or review. | *“…if you want longevity with the magazines …you have to take what they send you. They tend to send you what you’ve done before, so there’s very little renewal in the Reviewer’s frame of reference”* [C1] |
| Recognition | The critic feels a disconnect between the expected and actualized response to their work including issues around the payment, misunderstanding of their aims or meaning, and overly negative portray of critics. | *“And I’ve always said to people, ‘Look, everybody hates Critics. Get your machine guns out’”* [C8] |
| Market | Operating in the much larger field of published opinion and with multiple novel channels of communication (e.g. blogs, twitter, Amazon). | *“…the freeness of the Internet is a great boon in some ways, but it’s a disaster in others, because it’s overload, information overload …And, you know, it’s very difficult to weed out what opinions are worth reading, for readers”* [C4]  *“…we are mediators. Somewhere in-between a diffuse, heavily changing public”* [C12]  *“I don’t know how people are going to consume music. The question is if you’ve got YouTube and you’ve got iTunes and, you know, all these massive channels for acquiring music, how do you guide people and do people want to be guided?”* [C1] |
| Uncertain Future | Thoughts about changes in the industry, consumer habits, or his/her role. Often that led to concerns for the profession including the idea that it is losing volume and significance. | *“…I think record criticism is declining. It will probably dissolve”* [C10]  *“…the social structures and social mores have given people confidence to make their own decisions. So, the role of the Critic’s not necessary”* [C1]  *“…maybe the critical blog is the key, or maybe that’s a future manifestation of the Critic. And maybe what we have to look for is it’s how the roles are changing and what they’re going to metamorphise into”* [C1] |
| **Topics – Things I discuss** | | |
| Package | Elements of the recording that are extra to the actual sound, including the programme, composer, instrument and score edition used, sleeve notes, cover art design, comments by the artist, and issues of translation. | *“I will also definitely comment on the production of the CD and the quality of the sleeve notes and how well they are written and how well they’ve been translated”* [C3] |
| Recording Quality | The critic’s impression of the sound quality of the recording. It may include comments on the sound produced by the performer, only in reference to the nature of music recording and engineering. | *“I will certainly comment on the toned production of the Pianist, how it’s been recorded, the quality of the recording, the acoustic and whether …it sounds like a studio recording or whether …the artist had been able to transcend the recording studio and give me the impression that it’s a live important event, which is just happening”* [C3] |
| Performance Achievement | Aspects of the interpretation, including comments on style, originality, communication, and spontaneity as well as comments on musical parameters like tempo or phrasing. | *“…you’re saying how the performance is, what was good about the performance, you know, the expression and the phrasing”* [C4]  *“…these are important things to cover. The liveliness of the Gestaltung and of course the faithfulness to the text”* [C9] |
| Artist | General comments on the musician, their career, school they come from, track record of recordings, as well as their skills, including general comments on the musician’s technique. | *“…I would then offer a few background information. Biographical information of the interpreter, what has s/he done so far, to introduce the musician a bit”* [C12] |
| Place | Contextualization of the recording in terms of its history, its circumstances and its place in the emerging market. | *“I always like to contextualise a record, you know, when was it made? Why was it made? Who was it made for? What were the circumstances around the recording?”* [C1] |
| Response | Comments on the critic’s affective reaction to the music or evaluation related comments. | *“…when something catches your attention …then I usually put this at the beginning”* [C9] |
| Advice | A call to action for the reader, in terms of whether to buy or whether and how to engage with the recording. | *“…that was something as well that you’d want to consider in the review …you can …say, well, you might not want to listen to this all in, you know, in one go”* [C7]  *“Or to say, “Actually, don’t waste your money. There is a much better version, much cheaper, by a much better Pianist”* [C3] |
| **Tools – Things I use when writing** | | |
| Listening | Themes involved in the act of listening itself. |  |
| *Good Equipment* | The use of a high-quality reproduction system at least at some stage of listening. | *“I’ll come back and then I’ll listen to it again in my study on good equipment, proper CD player, proper speakers, that’s important”* [C3] |
| *Score* | To retain the score to hand as reference material while listening. It extends to comments on the work necessary to get hold of the score in the first place. | *“…I have the score of most works at home. That means I can listen to the works …with the score at hand”* [C13] |
| Feedback | Discussion with colleagues, editor, and/or the artist(s) during the review process. | *“…if a Reviewer comes back with a question about something, then I’ll try to listen as well so that I can, sort of, have an informed discussion about it”* [C7]  *“It also happens that I actually call the agency or the CD label and say: ‘I would like to briefly talk to the pianist. I simply have specific questions.’ …then we talk”* [C11] |
| Brevity | A concise writing style. Includes comments on the use of conciseness as a rhetoric tool. | *“…space is the crucial thing, always, with journalism. …if you’re only allowed a small slot, you must keep within that slot and it does limit what you can do”* [C2]  *“…this is a really small slot, a small space, and it requires conciseness”* [C10] |
| Comparison | The reviewer contrasts the recording to others or to related experiences, for example, seeing the performance live. | *“…of course, always put it in a context of other performances, talking about different approaches to it”* [C8]  *“A bad review considers the work or the CD as an individual object. …But a good one shows that this CD indeed does not stand alone, it is anchored in a wider space, in a repertoire”* [C14] |
| Justify | The critic gives reasons for his/her judgements and opinions. It includes comments on being factual and rational and on the use of examples as a means to back up a value judgement. | *“…it’s part of the information that you need to give, as to why you come to a particular conclusion about a disc. …I think that’s, perhaps, what the essence of all of this is about. …you have to justify and make clear your process of thinking”* [C5]  *“You argue. You reason, exemplify, and justify. This is critique”* [C11] |
| Language | Comments on the use of different vocabulary and linguistic styles to describe the recording. |  |
| *Specialized* | The use of musical terms (fermata, counterpoint, Leitmotiv). | *“I don’t use technical terms in reviews. Unless you actually have to, you know, there are times when you can’t get away without using a technical term”* [C6] |
| *Symbolic* | The use of figurative speech, for instance metaphors, similes, or personifications. | *“…metaphor and similes, and everything else can be useful and can colour your writing”* [C5]  *“…I don’t just always use picture, metaphors, because I think pictures should actually emerge from the music* [C9] |
| *Humor* | The writing aims to have an amusing or comical aspect. It includes comments on wit, satire, or irony. | *“I think it’s important at times to put a little humour into it”* [C3] |
| *Distance* | The use of first and third person. | *“…it’s interesting, I think, the use of whether you put yourself into the review or not. Whether you use I. I think this, or I think that, or whether you write it from a more detached point of view”* [C7] |
| Narrative Structure | The writing features recognizable story elements and devices including clear headline, distinct opening or closing statements or a core message / angle. This also extends to comments on the placement or order of the different elements of the narrative structure. | *“…begin with a fanfare. So get the reader’s attention. And end with a cadence, so you get the feeling at the end that, yes, this is the end of the review. We have come to a conclusion”* [C6] |
| Rating | The use of quantified evaluations, like numbers or stars. | *“…they have a much more careful grading system, five stars, …they’re much more precise”* [C1]  *“The star system simplifies things at times, but this simplification takes away the possibility to undergo very differentiated experiences”* [C12] |
| Thoroughness | Accuracy and diligence in the reporting of details about a recording. | *“…everyone does mistakes, I have, I think, Richard Strauß…, I have somehow confused double-S and sharp-S. And such a typo devalues the review unbelievably”* [C13] |
