# Supplementary material for: The critic’s voice: On the role and function of criticism of classical music recordings
Source: Front Psychol. 2022 Sep 29;13:925394. doi: 10.3389/fpsyg.2022.925394 (PMC9557232; doi:10.3389/fpsyg.2022.925394)
Supplement: Supplementary file 4 [file Table_4.docx]

## The Critic’s Voice Alessandri, Baldassarre & Williamson

## Supplementary Material 4 – German Quotes

**Original text of the German quotes used in the article.**

| *Result section* | *Theme* | *Quote* |
| --- | --- | --- |
| 1 Hats | Teacher: Audience | “…als Musikkritiker oder -kritikerin ist man heute auch Gesellschaftskritiker. Man ist heute Erzieher, …Pädagoge. Man hat pädagogische Aufgaben zu erfüllen” [C11] |
|  | Judge | “…das könnte schon eine Funktion sein ...der professionellen Kritik. …dass du einfach in diesem Dschungel ein paar Referenzpunkte hinsetzt, wo man sich darauf beziehen kann” [C9] |
| 2 Principles | Respect: Artist | “Grundsatz ist immer …den Menschen, der mir [die Aufnahme] bietet, immer ernst zu nehmen. Und das bedeutet für mich, dass ich mir überlege, was will er oder sie sagen“ [C11] |
|  | Interesting | “Sie müssen mit einem Text einen Leser auch gewinnen. Sie müssen ihn fesseln! Sie müssen ihn fangen” [C10] |
| 3 Challenges | Influence | “Es ist eine gewisse Angst …jemanden zu beleidigen oder zu verletzen” [C10] |
|  | Pressure: Industry | “Das geht mittlerweile soweit, dass die Plattenfirmen dort Druck ausüben, dass keine Verrisse mehr erscheinen sollen, und es erscheinen keine Verrisse mehr” [C12] |
|  | Market | “…wir sind Vermittler. Irgendwo zwischen einem diffusen sich ganz stark ändernden Publikum” [C12] |
|  | Uncertain Future | “…ich glaube, die Schallplattenkritik ist in einem Niedergang. Sie wird wahrscheinlich verschwinden” [C10] |
| 4 Topics | Performance Achievement | “…das sind wichtige Sachen, die drin kommen. Eine Lebendigkeit der Gestaltung, natürlich dann auch Texttreue” [C9] |
|  | Artist | “…da würde ich dann eher noch so ein paar Hintergründe liefern. Biografische Daten zu dem Interpreten, was hat der bisher schon gemacht, dass man eben auch diesen Musiker vielleicht mal ein bisschen vorstellt” [C12] |
|  | Response | “…wenn dich etwas anspringt in einer Aufnahme... Dann setz ich das oft an den Anfang” [C9] |
| 5 Tools | Listening: Score | “…die meisten Werke habe ich als Partitur zu Hause. Das heißt, ich kann mir die Werke auch nochmal mit Partitur anhören” [C13] |
|  | Feedback | “Das passiert auch, dass ich dann wirklich die Agentur anrufe oder das CD-Label und sage: ‚Ich möchte gerne mit der Pianistin oder mit dem Pianisten kurz telefonieren. Ich hab einfach gezielte Fragen’ …dann sprechen wir darüber” [C11] |
|  | Brevity | “Und das ist …sehr wenig also wenig Platz, wenig Raum, und das verlangt Konzentration” [C10] |
|  | Comparison | “Eine schlechte Kritik betrachtet das Werk oder die CD als singuläres Objekt. …Aber eine gute zeigt, dass diese CD eben nicht alleine dasteht, sondern dass sie eben in einem ganzen Areal von ...also im Repertoire eben …verankert ist” [C14] |
|  | Justify | “Man argumentiert. Man erörtert und erläutert und begründet. Das ist Kritik” [C11] |
|  | Language: Symbolic | “…ich gehe nicht einfach immer mit Bildern, mit Metaphern, weil ich finde, Bilder müssen eigentlich aus der Musik …entstehen” [C9] |
|  | Rating | “Das [Sternesystem] vereinfacht es manchmal, aber in dieser Vereinfachung …entäußert sich der Möglichkeit, selber sehr viel differenziertere Erfahrungen zu machen” [C12] |
|  | Thoroughness | “…jeder Mensch macht Fehler..., hab dann, glaube ich, Richard Strauß..., ich habe irgendwie Doppel-S und Eszett verwechselt. Und das entwertet natürlich …so ein Tippfehler im Prinzip …entwertet unglaublich die Kritik” [C13] |
